# Supplementary material for: WRKY Transcription Factors in Cassava Contribute to Regulation of Tolerance and Susceptibility to Cassava Mosaic Disease through Stress Responses
Source: Viruses. 2021 Sep 13;13(9):1820. doi: 10.3390/v13091820 (PMC8473359; doi:10.3390/v13091820)
Supplement: Supplementary file 1 [file viruses-13-01820-s001.zip › Final Supplementary Tables, Figures and Legends/Supplementary Figure Legends.pdf]

Supplementary Figure Legends

Figure S1 Predicted protein-protein interactions between AtWRKY homologs of differentially expressed MeWRKYs and their interacting partners in a central AtWRKY 33, and 53, 40 and 70 protein-protein network (**A**) T200 and (**B**) TME3 at 12 dpi. Networks were created using STRING v.11.

Figure S2 Predicted protein-protein interactions between AtWRKY homologs of differentially expressed MeWRKYs and their interacting partners in a central AtWRKY 33, and 53, 40 and 70 protein-protein network **(A**) T200 and (**B**) TME3 at 67 dpi. Networks were created using STRING v.11.

Figure S3 Upregulated gene partners in enriched hormone pathways associated with differentially expressed (up-regulated) AtWRKY homologs in **(A**) T200 at 12 dpi (**B**) T200 at 32 dpi (**C**) T200 at 67 dpi, and **(D**) TME3 at 32 dpi. Networks were visualised using Cytoscape v.3.6.1.

Figure S4 Resistance protein RPW8-NBS interacting protein partners identified using STRING v.11.
