# Supplementary material for: WRKY Transcription Factors in Cassava Contribute to Regulation of Tolerance and Susceptibility to Cassava Mosaic Disease through Stress Responses
Source: Viruses. 2021 Sep 13;13(9):1820. doi: 10.3390/v13091820 (PMC8473359; doi:10.3390/v13091820)
Supplement: Supplementary file 1 [file viruses-13-01820-s001.zip › Final Supplementary Tables, Figures and Legends/Supplementary Table Legends.pdf]

Supplementary Table Legends

Supplementary Table S1 Quantitative PCR [log2 relative change [ddc(t)] in WRKY expression and primer sequences.

Supplementary Table S2 Significant (1×10^{-8}) enriched GO terms (biological processes) from differentially expressed gene data sets in T200 and TME3 at 12, 32 and 67 days post *South African cassava mosaic* virus infection.

Supplementary Table S3 Differentially expressed (DE) interacting gene partners (and their GO functions) associated with *Arabidopsis* homologs (AtWRKY33 and 53 in T200 and AtWRKY40 and 70 in TME3) of upregulated *MeWRKY* TFs in enriched SA, ET, JA or ABA hormone pathways post-SACMV infection.

GOs associated with Arabidopsis homologs of differentially expressed (DE) MeWRKYs and their DE interacting gene partners in a central AtWRKY 33, 40, 53 and 70 protein-protein network.

Supplementary Table S4 AtWRKY28 (MeWRKY81 homolog) interacting partners and their functions.

Supplementary Table S5 Log2 fold expression of *AtWRKY* homologs in susceptible and resistant plant host-geminivirus infections at different stages of infection.
