# Supplementary figures and images for: WRKY Transcription Factors in Cassava Contribute to Regulation of Tolerance and Susceptibility to Cassava Mosaic Disease through Stress Responses
Source: Viruses. 2021 Sep 13;13(9):1820. doi: 10.3390/v13091820 (PMC8473359; doi:10.3390/v13091820)

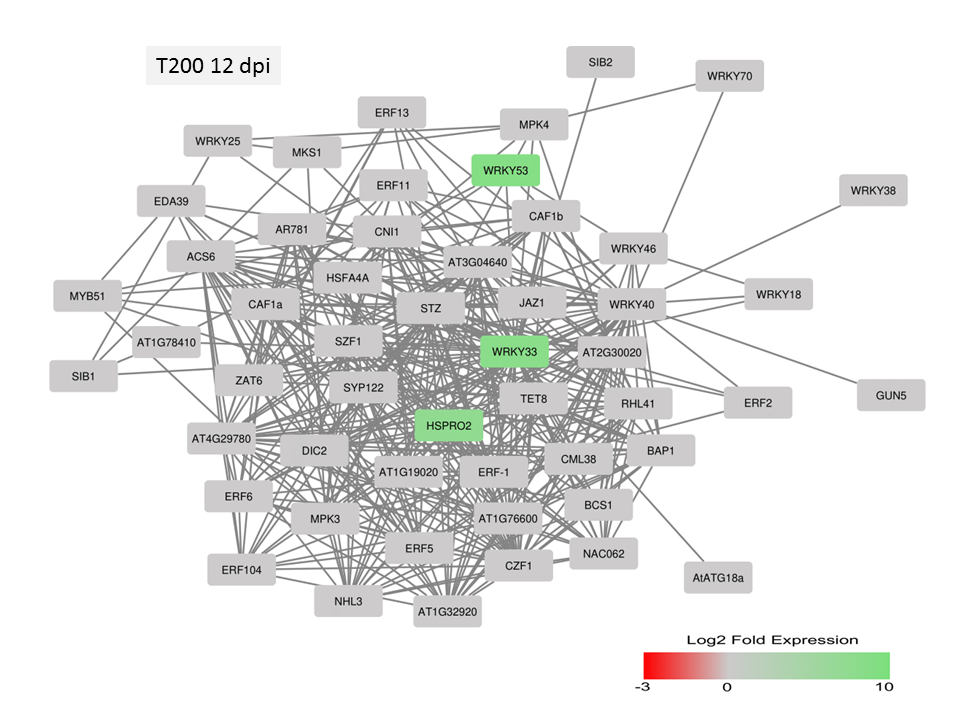

Supplement: Supplementary file 1 [file viruses-13-01820-s001.zip › Final Supplementary Tables, Figures and Legends/Figure S1A.png]

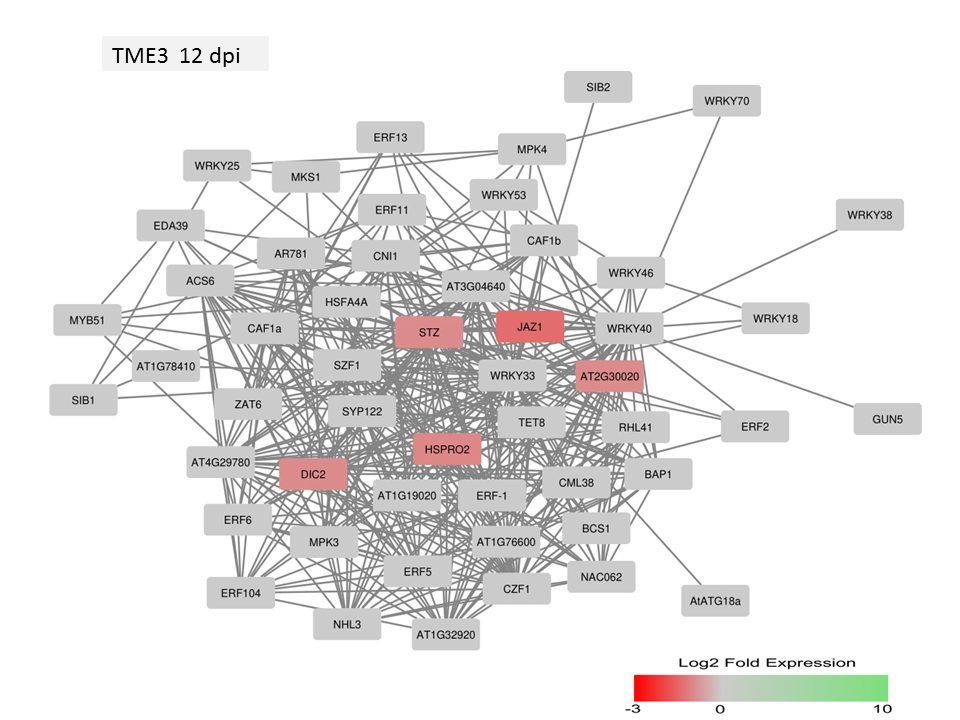

Supplement: Supplementary file 1 [file viruses-13-01820-s001.zip › Final Supplementary Tables, Figures and Legends/Figure S1B.png]

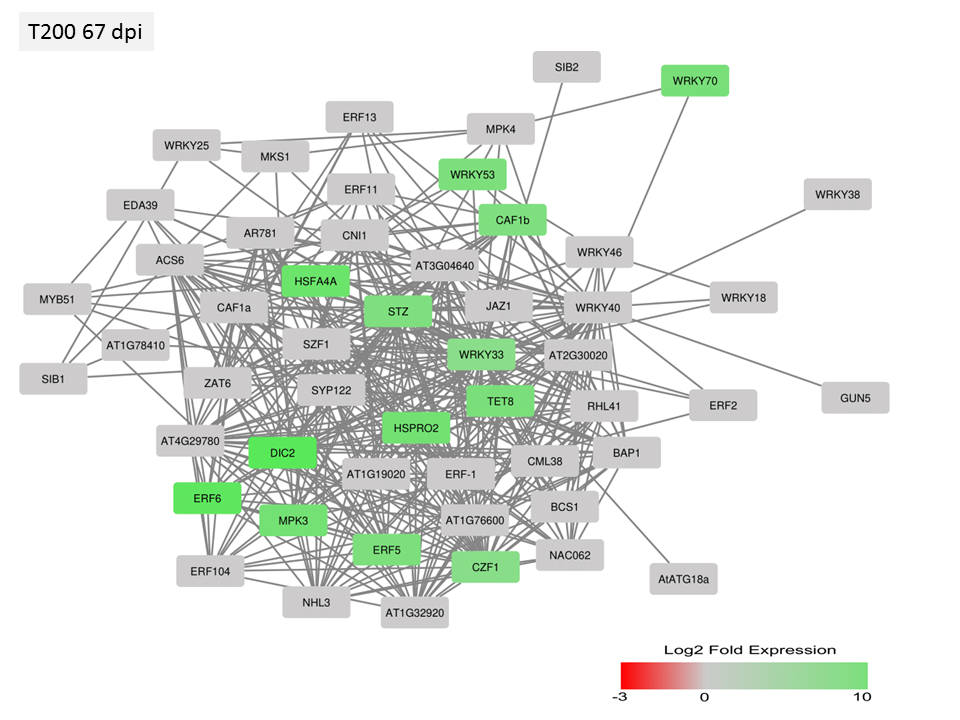

Supplement: Supplementary file 1 [file viruses-13-01820-s001.zip › Final Supplementary Tables, Figures and Legends/Figure S2A.png]

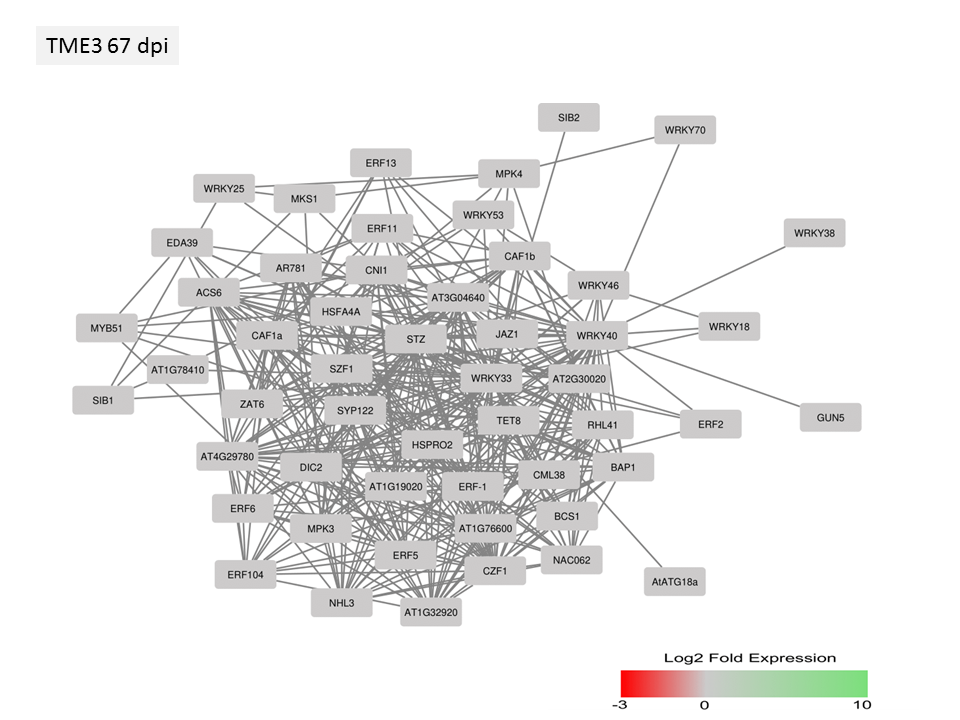

Supplement: Supplementary file 1 [file viruses-13-01820-s001.zip › Final Supplementary Tables, Figures and Legends/Figure S2B.png]

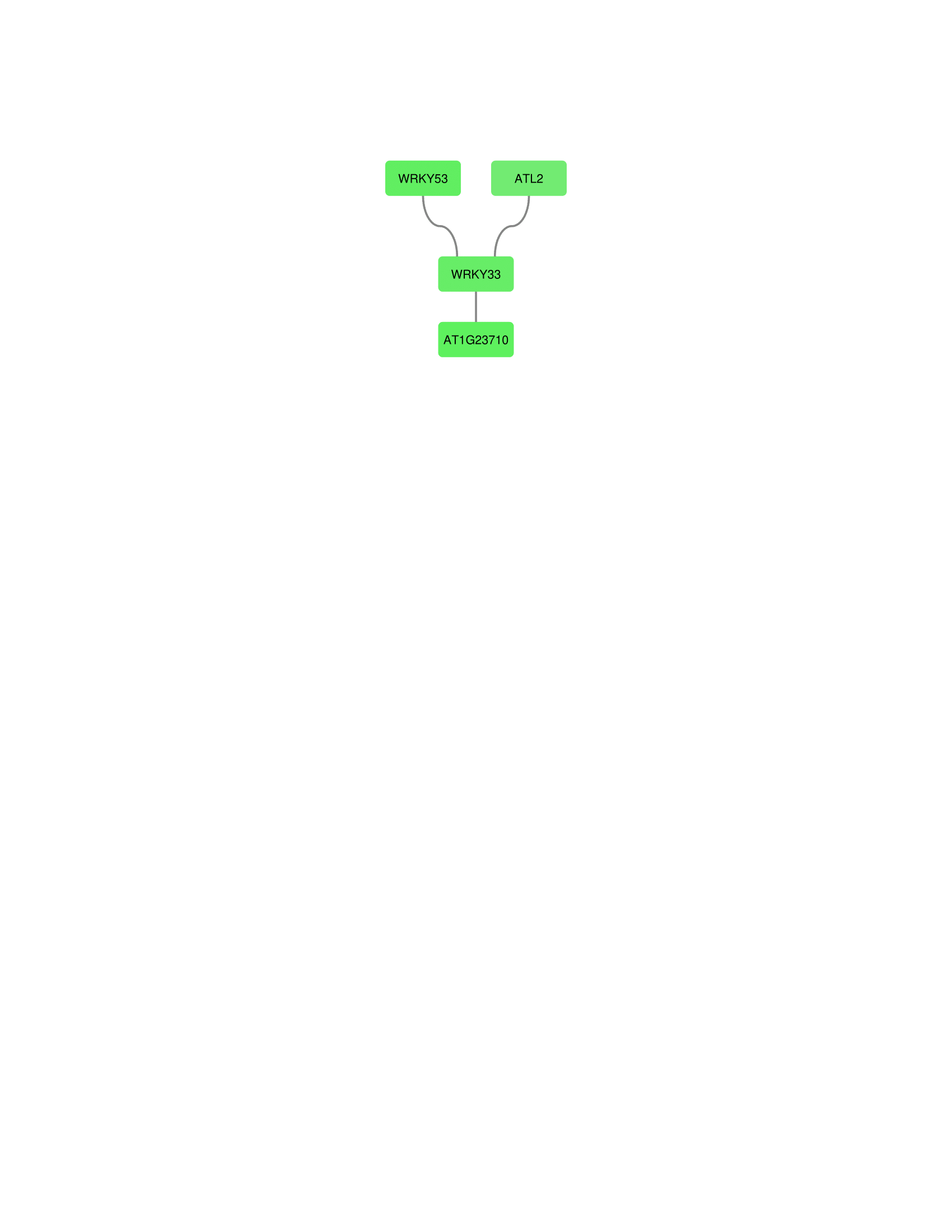

Supplement: Supplementary file 1 [file viruses-13-01820-s001.zip › Final Supplementary Tables, Figures and Legends/Figure S3A.png]

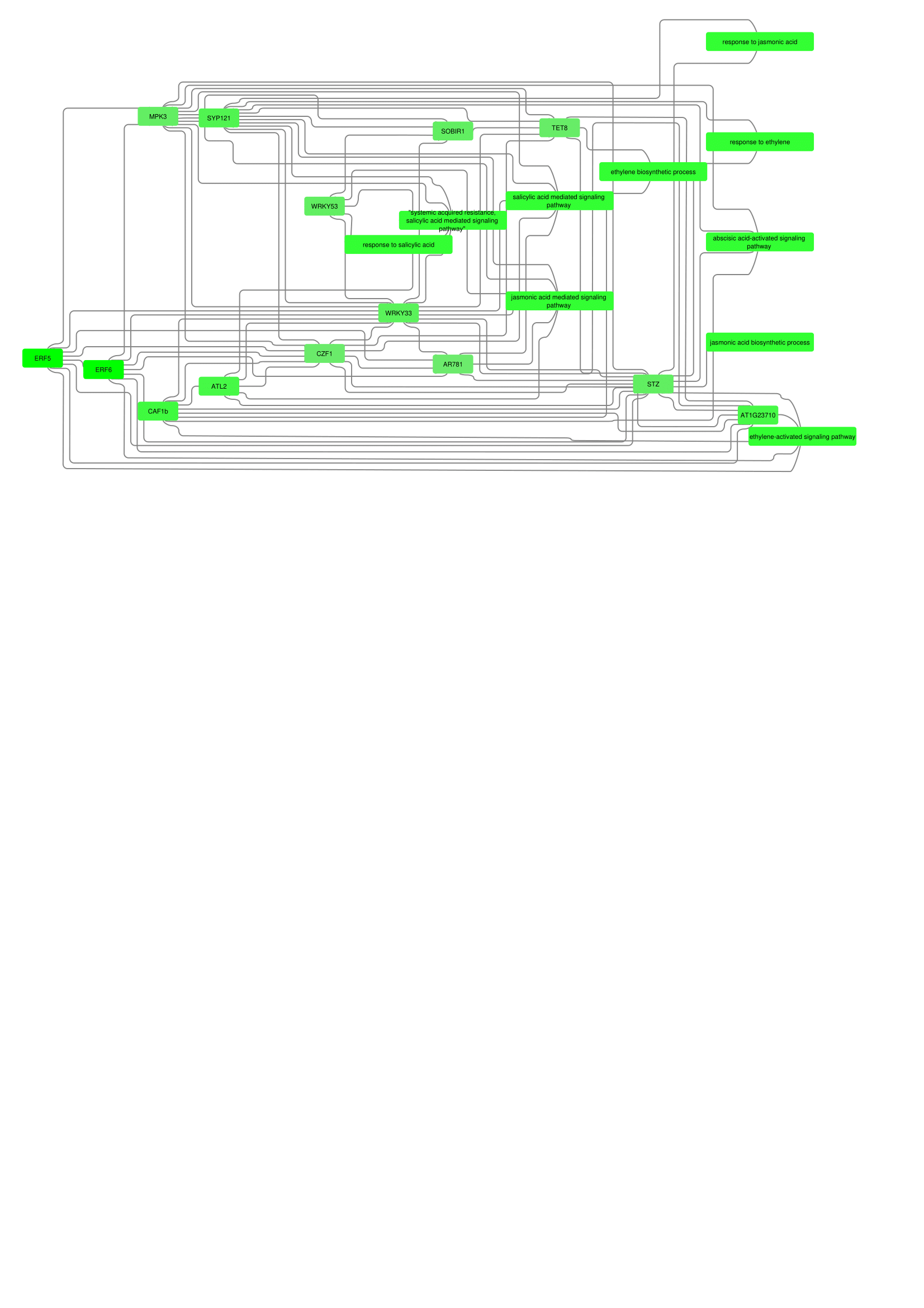

Supplement: Supplementary file 1 [file viruses-13-01820-s001.zip › Final Supplementary Tables, Figures and Legends/Figure S3B.png]

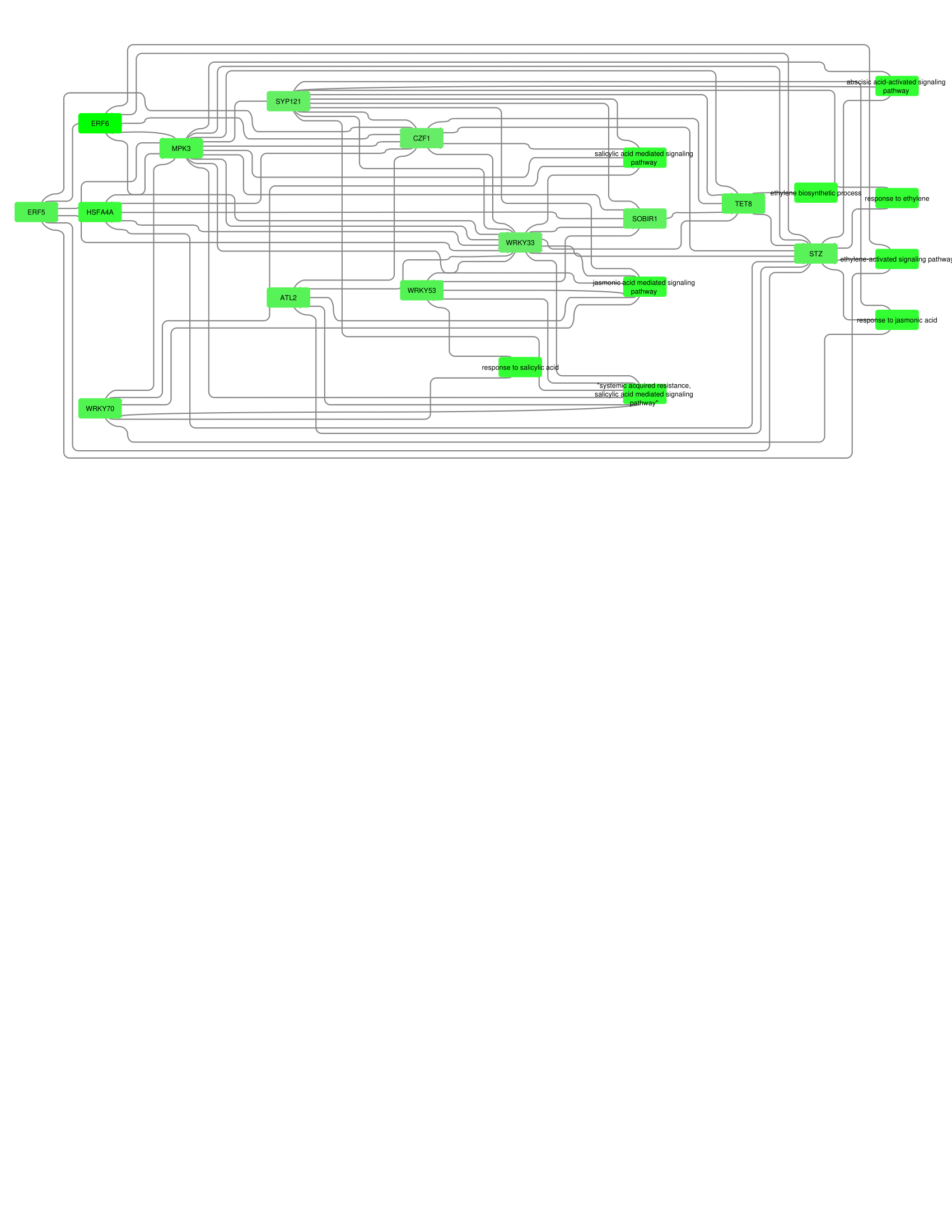

Supplement: Supplementary file 1 [file viruses-13-01820-s001.zip › Final Supplementary Tables, Figures and Legends/Figure S3C.png]

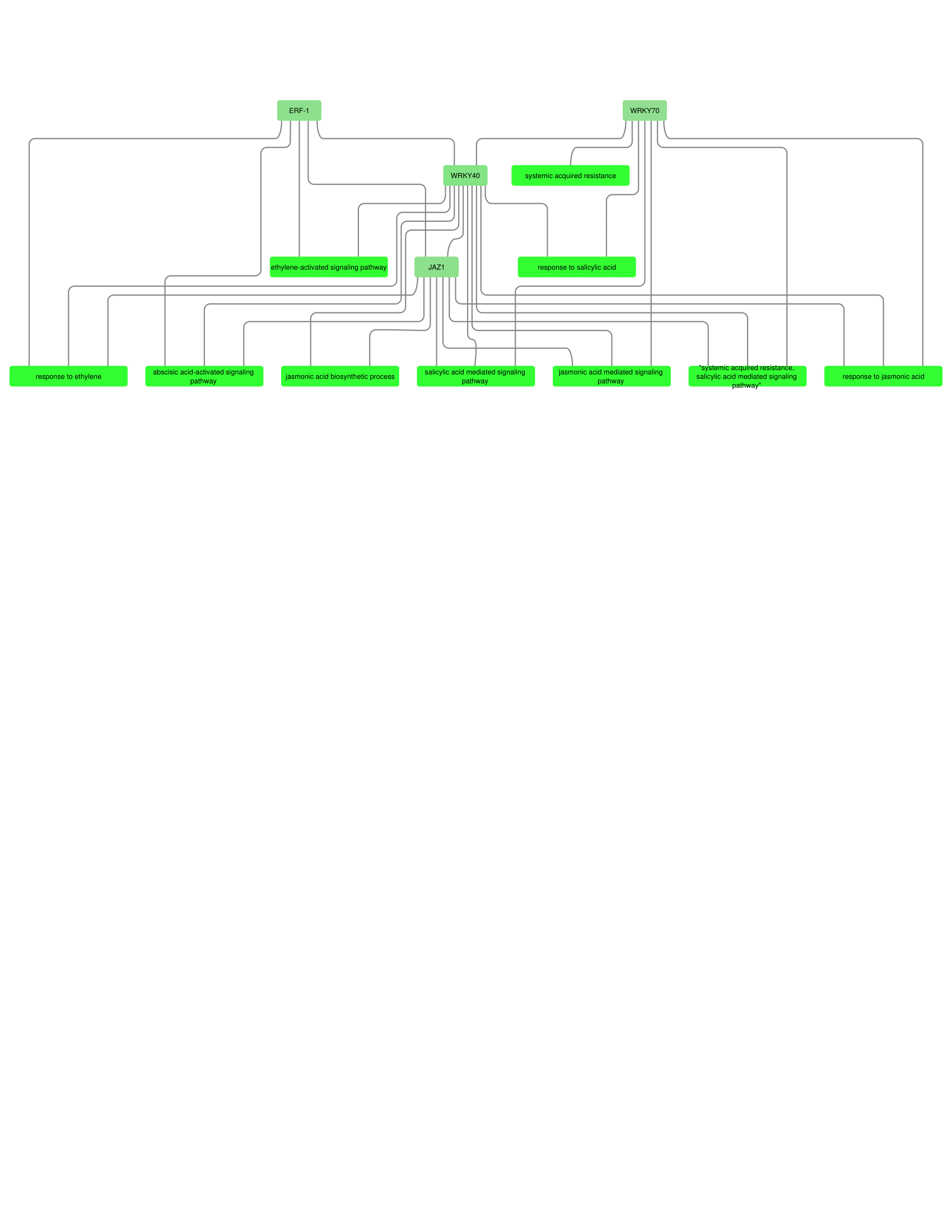

Supplement: Supplementary file 1 [file viruses-13-01820-s001.zip › Final Supplementary Tables, Figures and Legends/Figure S3D.png]

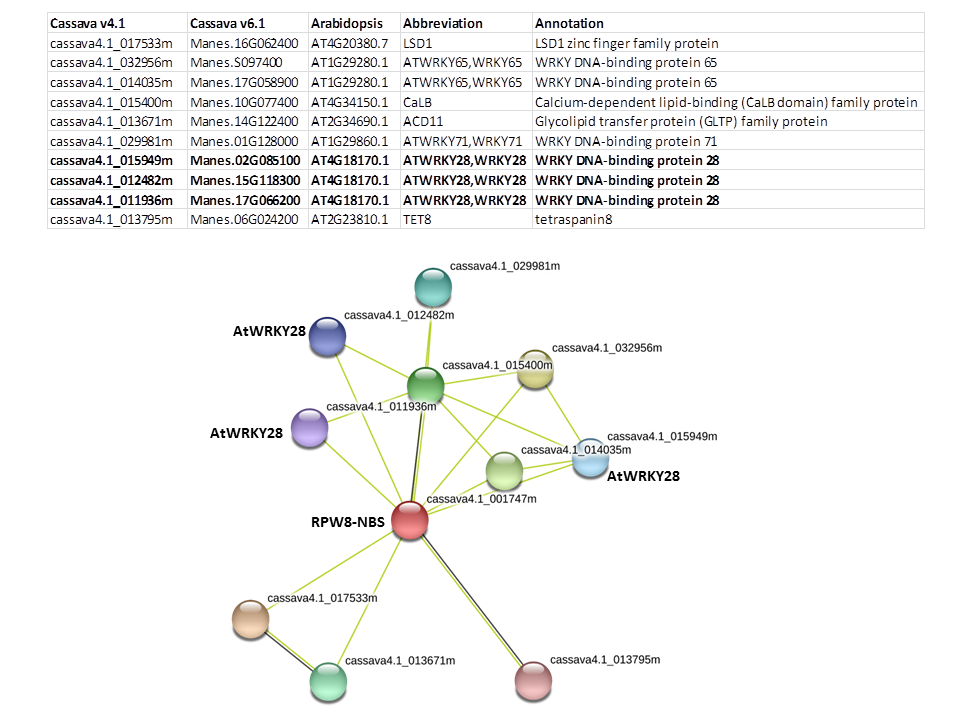

Supplement: Supplementary file 1 [file viruses-13-01820-s001.zip › Final Supplementary Tables, Figures and Legends/Figure S4.png]
